# Leaf Angle eXtractor: A high‐throughput image processing framework for leaf angle measurements in maize and sorghum

**DOI:** 10.1002/aps3.11385

**Published:** 2020-09-10

**Authors:** Sunil K. Kenchanmane Raju, Miles Adkins, Alex Enersen, Daniel Santana de Carvalho, Anthony J. Studer, Baskar Ganapathysubramanian, Patrick S. Schnable, James C. Schnable

**Affiliations:** ^1^ Center for Plant Science Innovation University of Nebraska–Lincoln Lincoln Nebraska USA; ^2^ Present address: Department of Plant Biology Michigan State University East Lansing Michigan USA; ^3^ Department of Mechanical Engineering Iowa State University Ames Iowa USA; ^4^ Present address: Department of Bioinformatics Federal University of Minas Gerais Belo Horizonte Minas Gerais Brazil; ^5^ Department of Crop Sciences University of Illinois Urbana Illinois USA; ^6^ Department of Agronomy Iowa State University Ames Iowa USA; ^7^ Department of Agronomy and Horticulture University of Nebraska–Lincoln Lincoln Nebraska USA

**Keywords:** computer vision, drought, image analysis, maize, phenotyping

## Abstract

**Premise:**

Maize yields have significantly increased over the past half‐century owing to advances in breeding and agronomic practices. Plants have been grown in increasingly higher densities due to changes in plant architecture resulting in plants with more upright leaves, which allows more efficient light interception for photosynthesis. Natural variation for leaf angle has been identified in maize and sorghum using multiple mapping populations. However, conventional phenotyping techniques for leaf angle are low throughput and labor intensive, and therefore hinder a mechanistic understanding of how the leaf angle of individual leaves changes over time in response to the environment.

**Methods:**

High‐throughput time series image data from water‐deprived maize (*Zea mays* subsp. *mays*) and sorghum (*Sorghum bicolor*) were obtained using battery‐powered time‐lapse cameras. A MATLAB‐based image processing framework, Leaf Angle eXtractor (LAX), was developed to extract and quantify leaf angles from images of maize and sorghum plants under drought conditions.

**Results:**

Leaf angle measurements showed differences in leaf responses to drought in maize and sorghum. Tracking leaf angle changes at intervals as short as one minute enabled distinguishing leaves that showed signs of wilting under water deprivation from other leaves on the same plant that did not show wilting during the same time period.

**Discussion:**

Automating leaf angle measurements using LAX makes it feasible to perform large‐scale experiments to evaluate, understand, and exploit the spatial and temporal variations in plant response to water limitations.

Over the past century, improvements in plant architecture and adaptation to higher planting densities have significantly increased maize grain yield. During this period, the average plant density per hectare has more than doubled due to changes in leaf angle that allow more efficient light capture (Duvick, 2005). Leaf angle is defined as the angle of the leaf blade relative to the center of the stalk as measured from a vertical line from the leaf/stem junction (Fig. 1). Genetic variation in leaf angle as a feature of a plant’s architecture influences canopy architecture and the efficiency of light capture for photosynthesis in maize and sorghum (Ku et al., 2010; Chen et al., 2015; Li et al., 2015; Truong et al., 2015). Although many traits influence canopy architecture, including the number of leaves produced per plant, leaf phyllotaxy, leaf length, and leaf width, one particular trait that contributes to canopy architecture—leaf angle—has been a particular focus of genetic investigation (Mickelson et al., 2002; Ku et al., 2010; Tian et al., 2011; Zhang et al., 2014; Mantilla‐Perez and Salas Fernandez, 2017). Under higher planting densities, a wide leaf angle increases leaf shading and negatively affects photosynthesis, whereas plants with narrow leaf angle architecture are able to intercept more light, thereby increasing grain yields (Pendleton et al., 1968; Lambert and Johnson, 1978). Maize hybrids with narrower leaf angles have yield advantages both under field conditions and in simulated studies (Duncan, 1971; Pepper et al., 1977). Natural variation for leaf architecture traits in maize and sorghum has been identified using biparental populations (Mickelson et al., 2002; Pelleschi et al., 2006; Ku et al., 2010; Truong et al., 2015; Dzievit et al., 2018; Tang et al., 2020), recombinant inbred lines (Li et al., 2015), and genome‐wide association studies (Tian et al., 2011), with quantitative trait loci overlapping candidate genes with known functions in leaf initiation, polarity, and leaf primordia development (Kerstetter et al., 1994; Moreno et al., 1997; McConnell et al., 2001; Juarez et al., 2004; Bolduc and Hake, 2009; Zhang et al., 2014). Introgression of alleles of two genes from the wild ancestor teosinte confers narrow leaf angle and enhances yields of modern maize hybrids grown at high densities (Tian et al., 2019).

**FIGURE 1:**
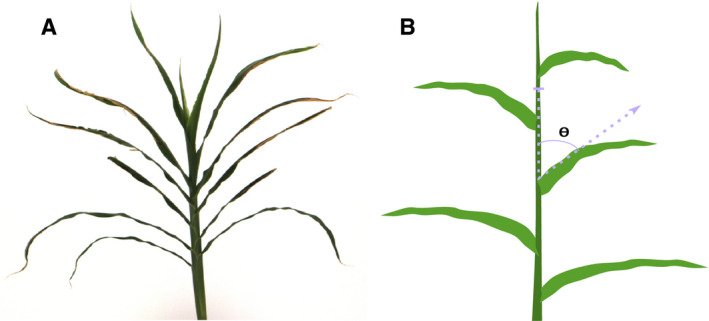
Leaf angle measurements from plant images. (A) Sorghum plant imaged on the LemnaTec phenotype analyzer. (B) Schematic representation of leaf angle determination, where leaf angle ‘ϴ’ is the angle made by the leaf blade with the stalk of the plant.

Advances in breeding and agronomic practices have led to steady growth in agricultural productivity, but increased weather variability threatens food security (Pryor et al., 2013; U.S. Global Change Research Program, 2018). Water limitation is one of the major abiotic stresses affecting growth, development, productivity, and geographical distribution of plants (Farhangfar et al., 2015). The ability of plants to survive and sustain yields under water‐limiting conditions encompasses a variety of adaptive mechanisms (Levitt, 1980). These include drought escape mechanisms such as rapid phenological development, adaptive plasticity, and remobilization of photosynthates, as well as avoidance mechanisms such as deeper rooting, reduced leaf conductance, and reduced leaf area via leaf rolling and/or movement (Beebe et al., 2013). The highly complex genetic architecture of drought response requires daily observation and measurement of noninvasive phenotypes (Eberius and Lima‐Guerra, 2009; Berger et al., 2010; Awada et al., 2018). The timing of measurements is critical because the impact of the water deficit depends heavily on the timing of the initiation of stress, the developmental stage of the plant, and the intensity of the applied stress (Wilkins et al., 2009; Dubois et al., 2017). Leaf rolling (i.e., leaf lamina rolls transversally to the midrib), wilting (defined by loss of rigidity due to diminished water content in the cells), and changes in leaf angle are among the most common drought response mechanisms in maize and sorghum (Farré and Faci, 2006). Genetic variation in these leaf characteristics has been studied in sorghum, particularly in the context of drought, where resistant varieties show more leaf curling than susceptible lines (Matthews et al., 1990; Farré and Faci, 2006). Narrower leaves in sorghum confer better adaptation to water deficit than seen for wider leaves in maize varieties (Farre, 1998). Thus, a comprehensive understanding of the genotype–phenotype relationship requires accurate phenotyping of these traits.

Conventional phenotyping tends to be labor intensive, expensive, and low throughput. Recent progress in plant phenotyping with the application of inter‐disciplinary technologies such as robotics, spectroscopy, and non‐invasive computer vision–based imaging have made it possible to measure plant performance nondestructively over an extended period (Eberius and Lima‐Guerra, 2009; Fahlgren et al., 2015). New phenotyping techniques should be high throughput, scalable across multiple platforms, and reduce the cost, time, and effort allocated to collecting trait data (Araus et al., 2018). While growth chamber–based platforms have the advantage of greater control, greenhouse‐ and field‐based platforms enable measurements of the whole plants in settings that more accurately mimic the target environments, thereby providing more biological value. However, while these automated platforms for measuring plant growth and development are now capable of collecting data at a faster pace, the extraction of useful measurement data from these raw images is not well developed. Hence, there is a need to develop new algorithms to process image data and extract biological information from difficult‐to‐measure traits.

Here, we report the development of a MATLAB‐based image‐processing framework for quantifying maize and sorghum leaf angles from image data. This framework, Leaf Angle eXtractor (LAX), was used to analyze data from two sets of experiments employing maize and sorghum plants exposed to water deficit stress. While the framework can, in principle, be applied to individual images, it provides the greatest time savings when employed for the rapid analysis of time‐lapse data with minimum additional user input. LAX made it possible to track leaf angle changes in individual plants at intervals as short as one minute under increasingly intense drought stress conditions. Given these results, LAX has the potential to serve as a valuable framework to analyze multiple genotypes for variations in leaf angle and to measure their responses to drought stress, and is particularly adept at tracking individual plants over time.

## MATERIALS AND METHODS

### Plant material and growth conditions

Three sets of plants, either maize or sorghum, were used to generate images for this study. The first set of plants (Set 1) comprised maize plants of the inbred line W22, grown in the greenhouse at the Donald Danforth Plant Science Center by the authors between 7 October 2013 and 20 November 2013. The greenhouse target conditions were 32/22°C day/night temperatures, 30% relative humidity, and a 16 : 8‐h photoperiod using supplemental metal halide lighting. The second set of plants (Set 2) were also maize (inbred line B73) grown at the University of Nebraska–Lincoln’s (UNL) automated phenotyping greenhouse under conditions described in Ge et al. (2016). The third set of plants (Set 3) included both maize (inbred line B73) and sorghum (inbred line BTx623) plants grown in the greenhouse facility of the UNL Beadle Center between 29 September 2017 and 22 November 2017. The greenhouse target conditions were 29/22°C day/night temperatures with a 16 : 8‐h photoperiod using supplemental illumination provided by LED lights with a target of 500–600 μM micromoles of photosynthetically active radiation per square meter per second. Plants were grown under well‐watered conditions for six weeks post‐sowing, and watering ceased three days before the beginning of imaging.

### Image data collection

Images for Set 1 were captured 44 days after planting using a first‐generation Raspberry Pi camera (1.5 megapixel [MP], Raspberry Pi Camera Module v1; Raspberry Pi Foundation, Cambridge, United Kingdom), and the image frames used for the initial development of the LAX framework were extracted from a compressed video file (Video S1) generated from the original series of still images collected by the Raspberry Pi camera. Set 2 plants were imaged using the automated greenhouse imaging system and RGB camera described in Ge et al. (2016). Set 3 plants were imaged 54 days after planting with a set of Bushnell 6‐MP Trophy Cams (Model 119636C; Bushnell Corporation, Overland Park, Kansas, USA). Cameras were set up approximately six feet away from each pot such that each plant’s axis of leaf phyllotaxy was facing the camera perpendicularly. Imaging was performed for eight days beginning at 5 a.m. (lights on) and continuing until 9 p.m. (lights off). The system used for greenhouse images is shown in Appendix S1.

### Image processing framework

**FIGURE 2:**
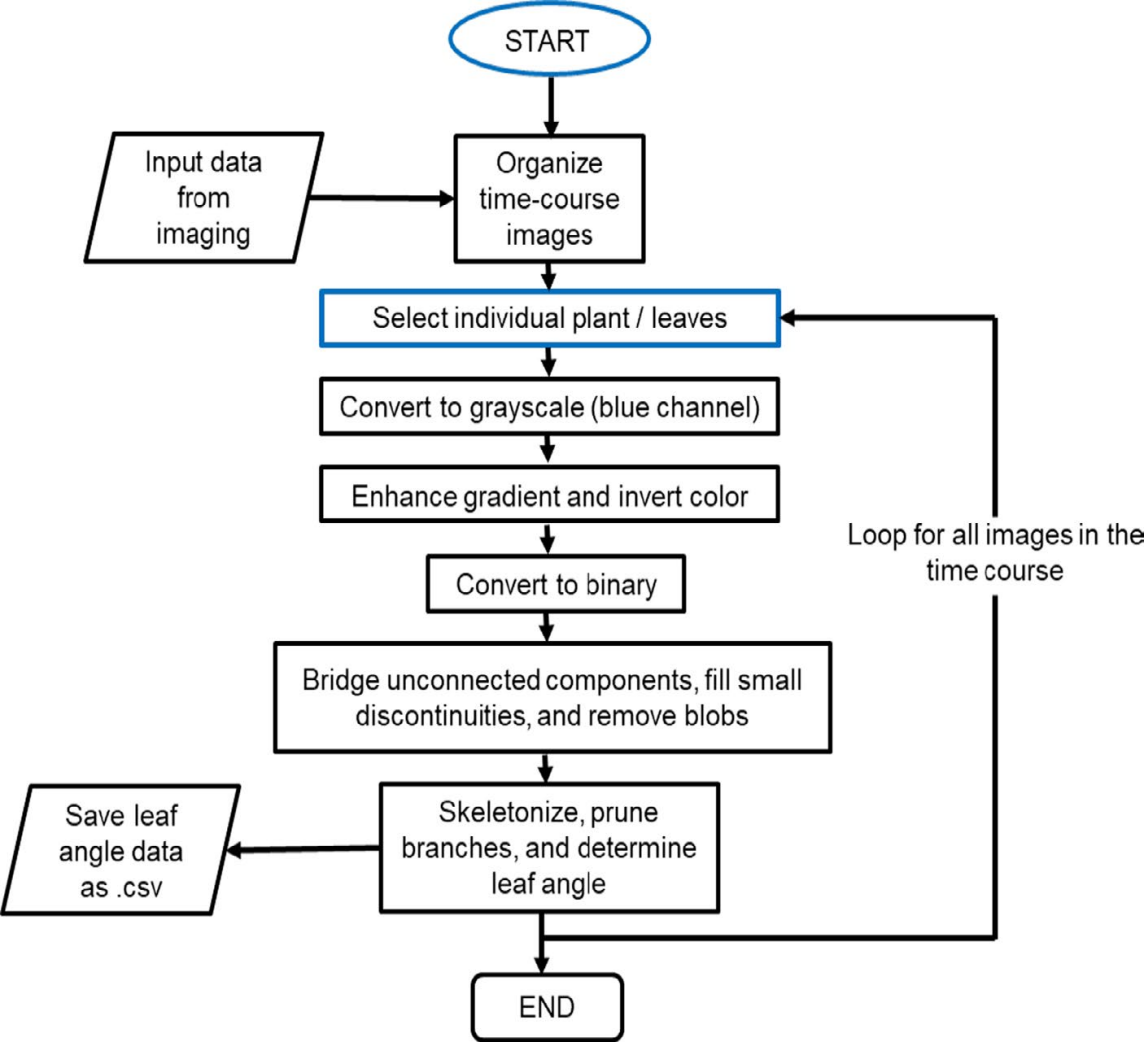
Flow chart showing how leaf angle estimation from images is conducted using the MATLAB framework.

An image processing framework was implemented in MATLAB R2014a (MathWorks, Natick, Massachusetts, USA) using Graphical User Interface Development Environment (GUIDE), a built‐in GUI editing utility (MathWorks, 1996). A flowchart describing the image processing workflow is shown in Fig. 2. All images of a series contained in a folder can be loaded to the LAX framework. User input is needed to select the first and the last image of the series.

#### Separation of foreground and background

Three‐channel RGB images were decomposed into three grayscale images. The grayscale image created from the blue channel was separated into foreground (plant) and background (everything else) using an intensity threshold. Otsu’s method (Otsu, 1979) was used to select a starting threshold value, with the results presented to the user for modification of the threshold value for approval.

#### Identifying a single plant and leaves of interest

User input is required to identify the plant stalk and leaves for leaf angle measurements. The user selects the stalk by placing a dot on the bottom center of the stalk, and then selects each leaf by placing a rectangular box close to the leaf–stalk junction without touching the stalk. The rectangular boxes are auto‐propagated to all the images in the series and will be considered for leaf angle measurements. These inputs help avoid background noise due to overlapping leaves that affect leaf angle measurements (Appendix S2A). The framework assumes that the stalk is vertical, which makes it suitable for maize and sorghum, which have stalks that are close to vertical in most genotypes with minimum noise (Bashyam, 2016).

#### Identifying leaf angle

Selected images are converted into grayscale images and sharpened using the ‘imsharpen’ function in MATLAB, which uses unsharp masking where an image is sharpened through subtraction of a blurred version of the image from itself. The image is complemented using ‘imcomplement’ to reverse the black and white pixels within the image to make the plant an active object. The ‘imcomplement’ function subtracts each pixel value from the maximum pixel value supported by the class and outputs an image based on the difference in pixel values. The modified grayscale image is converted to binary using ‘im2bw’, which replaces all pixels with the value ‘1’ if the luminance is greater than the provided level, and replaces all other pixels with the value ‘0’ (Appendix S2B–E). MATLAB version 2016 and above recommend using ‘imbinarize’ instead of ‘im2bw’. The image is then skeletonized, which reduces the thickness of the foreground object as much as possible while preserving connectivity. Angle data are extracted from the created line using ‘bwlabel’ to identify each region and ‘regionprops’ to find the angle of the region based on its ellipse (Appendix S2F–J). The function ‘bwlabel’ extracts labels for connected objects from the 2D binary image. Pixels are considered connected if either their edges or corners are touching when using 8‐connected pixel connectivity in ‘bwlabel’. The MATLAB function ‘regionprops’ measures the set of properties for each connected component in the binary image, which is done by fitting the smallest ellipse around the area identified as the leaf and extracting the angle made by the major axis of the ellipse with the vertical. This method utilizes all the leaf data (all of the connected component area) rather than reducing the useful data to a single pixel‐thick line, which would be more susceptible to error. Leaf angle is defined as the angle (in degrees) made by the leaf blade from a vertical line ascending from the leaf/stem junction (Fig. 1B). A lower angle indicates narrow leaves, whereas a higher angle represents wider leaves. If a foreign object blocks the leaf in any image, the leaf angle value will be empty and indicated by ‘NaN’. The LAX framework generates a plot of the leaf angle as output and provides the option to export the leaf angle data as a comma‐separated value (.csv) file.

To make this framework more accessible to researchers, a GUI was built into the application allowing for an intuitive interaction between the user and the software. The GUI instructs the user on each step of the operation and supplies confirmation of successful operations, thereby separating the end‐user from the underlying math, image processing, and computational processes.

### Ground truth measurements and validation

Validation was performed through a comparison of the angle measurements generated by the image processing software described above and manual measurements of the same images. Ten images were selected randomly for each of six plants, and for each plant the angles of the same three to five leaves were manually measured using a ruler and protractor across all 10 images, resulting in a total of 240 manual measurements of leaf angle.

## RESULTS

We developed a MATLAB software package for measuring leaf angles from large stacks of time‐series images collected from a single viewpoint using maize plants from Set 1 (see Methods). The basic organizational structure of the approach is illustrated in Fig. 2, and the stepwise processing of images for leaf angle analysis is shown in Appendix S2. Detailed documentation on the use of this software is provided in Appendix 1.

### Development of the image processing framework for leaf angle analysis

**FIGURE 3:**
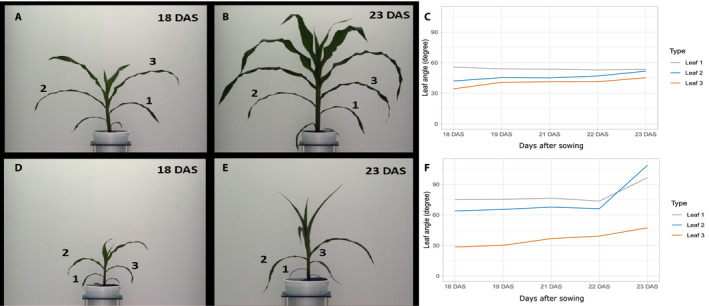
Leaf angle measurements during water deprivation in maize using images from Ge et al. (2016). (A, B) Maize genotype B73 grown under well‐watered conditions, 18 days after sowing (A) and 23 days after sowing (B). (C) LAX leaf angle measurements for three leaves of maize plants grown under well‐watered conditions. (D, E) Maize genotype B73 grown under water‐limiting conditions for 18 days after sowing (D) and 23 days after sowing (E), respectively. Water was stopped after 10 days after sowing. (F) LAX leaf angle measurements for three leaves of a maize plant under water‐deprived growth conditions. DAS, days after sowing. Numbers 1–3 identify the leaves measured in the study.

Previously published image data from a maize drought stress experiment conducted in an automated phenotyping greenhouse using the LemnaTec conveyor belt imaging system (LemnaTec GmbH, Aachen, Germany) were used to capture leaf angle changes (Ge et al., 2016; Liang et al., 2018). Plants from the study by Liang et al. (2018) were only imaged once per day, producing more substantial image‐to‐image changes in morphology and organ position. Three replicates each for well‐watered and water‐stressed B73 maize plants were selected from Set 3, and leaf angles were measured with LAX beginning eight days after drought initiation for five consecutive days to capture leaf wilting in water‐stressed plants. Plants displayed apparent differences in plant biomass and height under well‐watered and water‐stressed conditions (Fig. 3A, B, D, E) in agreement with the differences reported in Ge et al. (2016) and Liang et al. (2018). Drought‐induced leaf wilting was visible consistently in all three stressed replicates on the fifth day of imaging (Fig. 3D, E). LAX was able to show changes in leaf angle, coinciding with leaf wilting, whereas plants grown under well‐watered conditions did not show changes in leaf angle (Fig. 3C, E).

Six‐week‐old maize and sorghum plants from Set 3 were grown in the greenhouse under unstressed conditions and imaged every minute for eight days, starting three days after the cessation of watering. Leaf angle measurements were recorded for two to five leaves per plant. Only those leaves with a clearly visible leaf–stem junction were employed for leaf angle measurements using LAX. The exact number of leaves per plant was determined by the availability of mature and fully extended leaves. Over a nine‐day period, a total of 35,640 images (660 images each per day) were obtained from six plants.

### Evaluating the accuracy of LAX‐based semi‐automated measurements of leaf angle

Ground truth data were generated by manual measurement of apparent leaf angles in a random sample of 240 leaf/photo/plant combinations to evaluate the accuracy of leaf angle measurements generated by the LAX framework. Pearson correlation coefficients (*r* values) for LAX measurements and ground truth data for sorghum and maize were 0.82 (*P* < 0.001) and 0.83 (*P* < 0.001), respectively (Appendix S3).

### Leaf angle measurements for maize and sorghum under drought stress

**FIGURE 4:**
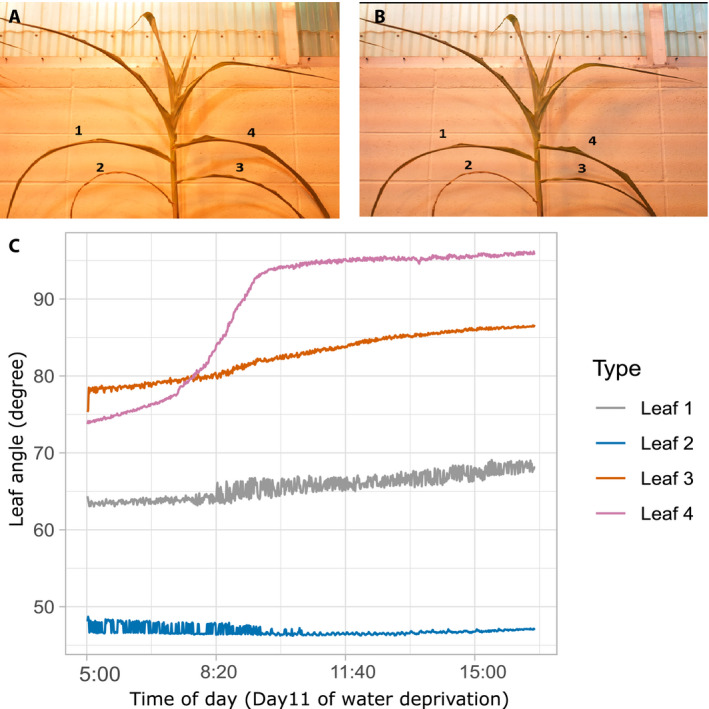
Plotting leaf angle changes during the day using 6‐MP cameras. (A, B) Images of maize plant M1 on day 11 of water deprivation. Leaves 1, 2, 3, and 4 are marked. (A) Image taken when the artificial lights in the greenhouse were turned on at 5 a.m. (B) Image taken the same day at 4:20 p.m. (C) Plot showing leaf angle changes of the four marked leaves during the day from 5 a.m. to 4:20 p.m. Note that the increased variation in leaves 1 and 2 is likely caused by wind blowing from the greenhouse cooling fans.

LAX leaf angle data for six‐week‐old maize and sorghum plants (from Set 3) under water‐stressed conditions were used to track changes in leaf angle over the course of the experiment. Consistent with previous reports, under non‐stress conditions sorghum leaves exhibited more erect leaf angles than maize (Appendix S4) (Flénet et al., 1996). Although sorghum plants showed leaf rolling symptoms by day 11, they did not show any signs of leaf wilting (Appendix S4C), whereas all maize plants exhibited leaf rolling by day nine and wilting by day 11 (Appendix S4E, F). In maize plants, wilting was visible from the plots of leaf angle as it showed a distinct increase in leaf angle, suggesting wilting of leaves during day 11 since the cessation of watering (Fig. 4). In all three maize B73 replicates, the uppermost leaf with a clearly visible collar showed wilting while the other leaves did not exhibit drastic changes in leaf angle (Fig. 4; Appendix S5). Thus, our LAX framework was able to clearly distinguish leaves that exhibited wilting from those leaves on the same plant that did not show wilting during the same time period (Fig. 4).

## DISCUSSION

Understanding the molecular mechanisms underlying complex traits such as drought response is challenging due to the manifestation of a multitude of physiological changes. Some of these stress phenotypes are easily measured, and their molecular mechanisms are better understood, while more complex phenotypes such as leaf angle measurements require advances in phenotyping techniques and data extraction to link phenotypes to genetic variation. This study reports the development of a MATLAB‐based framework (LAX) and its application to efficiently extract leaf angle measurements from time‐course image data showing leaf wilting during drought stress in maize and sorghum using both an automated greenhouse imaging system and the more portable and much lower‐cost system based on Bushnell 6‐MP Trophy Cams.

Dynamic traits such as stress responses change with time and environment, thus requiring repeated measurements over the course of an experiment (Awada et al., 2018). Maize hybrids with similar leaf water potential (ψL) under well‐watered conditions tend to show differential ψL responses (Lorens et al., 1987) and related visual symptoms such as leaf wilting (O’Toole and Cruz, 1980) when deprived of water. Although these temporal variations are critical for developing stress‐tolerant lines, measuring these variations among diverse genotypes is challenging due to the nature and amount of data that must be collected and analyzed. The LAX framework was implemented to automate leaf angle measurements from time‐course image data, enabling the large‐scale experiments that are needed to uncover variation in plant responses to adverse environmental conditions. LAX successfully measured changes in leaf angles in maize plants under different watering regimens using LemnaTec images from a previous study (Liang et al., 2018). Although LAX was able to track the timing of increases in leaf angle in response to drought stress, it was challenging to automate the framework for LemnaTec images because the plants were moved on conveyor belts, and plants sometimes rotated within their pot holders, resulting in changes to the orientation of the plant relative to the camera. Another consideration is that pictures should be taken when the plants are at a slower growth rate because rapid growth during the imaging time frame makes it difficult to index leaves in the time‐series images. Despite its utility as a robust phenotyping system, LemnaTec high‐throughput systems are expensive, thus they are not prevalent among plant breeding labs and are generally not available in the developing world. To adapt the LAX framework for cheaper alternatives of high‐throughput imaging, we analyzed thousands of images from standard 6‐MP cameras set up to image plants every minute; this allowed thousands of pictures to be taken of each plant (16 h × 60 images/h = 960 images in a day). In this study, all images of a series were loaded onto the framework without any memory issues. However, larger data sets may require implementing sequential loading of images. This time‐series image data provided a perfect setup to track changes in plant architecture at a much finer time scale of hours and can be used to understand both spatial and temporal responses of plants to drought stress. Consistent with previous reports of differences in drought response between maize and sorghum (Erdei and Taleisnik, 1993; Nagy et al., 1995; Erdei et al., 1996; Farré and Faci, 2006), maize leaves showed leaf rolling and wilting as the water deficit intensified, whereas sorghum leaves showed delayed leaf rolling. Our study was limited in power to dissect fine spatial details due to a limited number of replicates, but future work with more replicates will benefit our understanding of how leaves in different parts of a plant and at different developmental stages respond to water limitation.

Another limitation of the study is that it was not possible to completely automate the process. User input is required in the initial steps to identify the plant and to manually constrain the search area for a leaf of interest as the suboptimal conditions make it extremely difficult to identify and separate leaves. Whole plant skeletonization approaches have also been explored to track leaf traits including leaf angle (Bashyam, 2016). These approaches can work well so long as no leaves intersect in the 2D photo taken of the plant. These crossovers, which are quite common in mature maize or sorghum plants, although less common in seedlings, substantially reduce the accuracy of current skeletonization‐based approaches (Das Choudhury et al., 2018). LAX accepts the tradeoff of requiring user input once per image stack in order to be able to track changes in leaf angle even if there is leaf crossover between the leaf tip and the leaf stem junction.

Correlations between LAX‐based leaf angle measurements and ground truth measurements are slightly lower than in previous studies correlating image‐based phenotypic data and ground truth measurements (Gehan et al., 2017; Liang et al., 2018). Liang et al. have demonstrated that some traits show higher correlation than others; for example, plant height measured from image‐based phenotyping showed a higher correlation with ground truth measurements than plant biomass with its ground truth data (Liang et al., 2018). Although ground truth measurements for leaf angle were taken from pictures rather than measuring live plants in the field, the observed high correlation between these measurements and LAX output will allow researchers to estimate leaf angle measurements for large numbers of samples. LAX immensely reduces the time spent in measuring leaf angle in greenhouse‐based studies where the same plant is imaged repeatedly. It should be noted that the LAX framework is optimized for tracking changes in leaf angles in individual plants over time and is not recommended for comparing leaf angle differences between genotypes due to the possibility of bias introduced between batches of images as a result of differences in plant architecture and imaging. In comparisons across different plants, potential bias can be introduced by differences in phyllotaxy, which result in individual leaves sometimes being less than perfectly perpendicular to the camera. However, future studies on larger populations of inbred and hybrid lines are necessary to reveal genotypic and spatio‐temporal differences in leaf wilting across multiple abiotic stressors. These advances in high‐throughput leaf angle measurements will not only be critical in understanding plant response to water limitation but also assist breeders in tapping this diversity for the development of hybrids with drought tolerance and ideal ideotypes for future increases in planting densities.

## Supporting information


**APPENDIX** **S1.** Camera setup to acquire time‐course images from maize and sorghum plants under water deprivation. (A) Image showing the actual 6‐MP camera setup at the Beadle Greenhouses, University of Nebraska–Lincoln. (B) Illustration showing the distance between the camera and the pots, and the perpendicular angle made by the axis of the leaf phyllotaxy with the face of the camera.

**APPENDIX S2.** Stepwise progression of image processing to obtain leaf angle measurements from plant images. (A) Individual plant selected for leaf angle analysis. (B) Image converted to grayscale (in this particular case, blue channel). (C) Enhancing gradient. (D) Inverting color of the picture. (E) Image converted to binary. (F) Image thickened and stalk of the plant emphasized. (G) Unconnected components are bridged, small discontinuities corrected, and image blobs removed. (H) Skeletonized image. (I) Branches are pruned. (J) Determination of leaf angle from the processed image. (Example images shown here are from maize plants grown in the Donald Danforth Plant Science Center, St. Louis, during 2013.)

**APPENDIX S3.** Average difference between leaf angle measured using LAX and ground truth measurements.

**APPENDIX S4.** Differential phenotypic response to drought stress in sorghum (A–C) and maize (D–F). (A) Six‐week‐old sorghum plant (Btx623). (B) Sorghum plant nine days after water deprivation. (C) Sorghum plant showing leaf rolling symptoms 11 days after water deprivation. (D) Six‐week‐old maize plant (B73). (E) Maize plant nine days after water deprivation showing signs of leaf rolling. (F) Maize plant showing leaf wilting after 11 days of water deprivation.

**APPENDIX S5.** Consistent wilting of the upper leaf with clearly visible collar across replicates. (A–C) Maize plant 9‐M2, showing wilting of leaf after day 11 of water deprivation. (D–F) Maize plant 10‐M3, showing wilting of the same leaf after day 11 of water deprivation. The blue arrow indicates the leaf that showed wilting in both maize replicate through the time‐course images.

**APPENDIX S6.** Sample screens showing LAX framework usage. (A) Graphical user interface (GUI) welcome screen for the LAX framework obtained after running new_wilt_gui.m function in MATLAB. (B) Selection of the first image in the series. (All images belonging to a series stored in a folder can be loaded at once.) (C) Selecting the stalk of the plant by clicking the cursor at the center of the plant stalk. (D) Adjusting the width and the height of the plant image suitable for leaf angle measurements. (E) Selection of leaves for leaf angle measurement. Rectangles must be drawn close to the leaf–stalk junction without touching the stalk. Care must be taken while drawing the rectangle so that the entire change in the leaf angle can be captured in the drawn rectangle (as shown in H). (F) Leaf angle measurements will be recorded for as many leaves as selected by the user. (G) Thresholding can be adjusted by moving the slider or inputting threshold values. (H) When thresholding is completed for the last image in the series, the screen shows both the first and the last image and the rectangles drawn to select the leaves. (I) Clicking ‘Start analysis’ begins the analysis of each leaf for angle measurements. (J) The final screen shows the plant image with rectangle boxes and leaf number. Clicking the ‘Export Data’ icon at the bottom outputs leaf angle measurements for the selected leaves as a .csv file.

**VIDEO S1.** Time‐lapse video showing the drop of maize leaves in response to water deficit stress over a single day. This video is also available at https://vimeo.com/256137800.

## Data Availability

The source code and GUI interface are available at https://github.com/Kenchanmane‐Raju/Leaf‐Angle‐eXtractor.

## APPENDIX 1 Detailed description of how to use the Leaf Angle eXtractor (LAX) image processing framework. Appendix S6 shows screenshots of each step performed while measuring the leaf angle from images.

### Welcome screen

The welcome screen serves as an initial starting point in the application, providing the user with useful information about the creation of the project as well as example imagery of the capability of the program. The user selects ‘New Project’ to continue to the next step. The welcome screen also has an ‘About’ button (question mark icon) that provides useful information about the program. The ‘New’ button will bring the user back to the welcome screen at any time.

### Selecting time‐lapse images

To define the set of images that make up the time‐lapse sequence, the user chooses a folder with all the images in the series. (If the pictures have arbitrary file names, then a sort function is used to order the files.) LAX sorts the images by their date of creation. The utility then stores all the images in a cell array, where each cell contains the RGB value data for a sequential image from the time‐lapse sequence. The user can then press the ‘Continue’ button to proceed to the next step or the ‘Undo’ button to return to the welcome screen.

### Selecting the initial frame

To pick the start frame for analysis, the user must choose an initial value by moving the slider. As the slider is moved, a preview of that frame is generated in the GUI. By pressing ‘Continue’, the slider value is retained for future use.

### Selecting a plant for analysis

Following the selection of the first frame, the user must specify the individual plant to be used in the analysis. This is accomplished by interpreting the location at which the user clicks the screen. The user can crop an individual plant by clicking the bottom center of the plant at the center of the stalk.

### Selecting leaves for analysis

Once the individual plant is selected, the user can choose the appropriate leaf or leaves for analysis. This enables the user to choose leaf angle data from leaves of interest, rather than collecting data for all leaves in the plant. For this, a rectangular selection tool is provided. Each rectangle selected by the user represents the search limits for a leaf during the analysis portion of the framework. To allow the user as much flexibility as possible, an added confirmation box is included after drawing a rectangle. This ensures that the user is able to redraw a rectangle should their initial choice be suboptimal.

### Setting the initial threshold

The user must define the thresholding properties to create a binary image. To accomplish this, the user can use the slider to adjust the threshold level. A manually editable text box that displays the slider value is also incorporated to change the threshold level. To switch between the red, green, and blue channels, a pop‐up menu selector is utilized. Finally, to allow functionality for infrared imagery, an ‘Invert Colors’ checkbox is added that allows the user to switch the criteria of the foreground from high intensity to low intensity. Pressing the ‘Continue’ button saves these properties and takes the user to the next step.

### Selecting the final frame

For the selection of the final frame, the preview image contains only the area within the original crop bounds rather than the entirety of the time‐lapse image frame. The sequential value of the first frame limits the lower bound for the final frame selection. The maximum value is constrained by the total number of frames in the time‐lapse sequence. Pressing ‘Continue’ retains the slider value and takes the user to the next step.

### Setting the final threshold

A final threshold step is utilized to accommodate a linear change in the lighting of the time‐lapse sequence. By having two different threshold levels, it is possible to interpolate level values for all frames under analysis, giving the user more control over the accuracy of the analysis. The final threshold step is performed on the last sequential image and is similar to the initial threshold step but has fewer variable objects because the color channel and the image inversion has already been determined. Pressing ‘Continue’ saves the final threshold level and brings the user to the ready screen to perform analysis.

### Performing leaf angle analysis

This step serves as a confirmation screen before performing the leaf angle analysis. It displays the binary image of the initial and final frames side by side. By doing this, the user can correct any possible errors made during the initial inputs before the analysis is underway. Pressing the ‘Start Analysis’ button begins the estimation of leaf angle. The analysis is completed for each leaf of the plant by isolating the region of the leaf, thresholding the image, and using the region property of the largest connected component to determine the orientation of the leaf. A status bar is utilized to inform the user of the progression of the analysis. After the analysis is complete, the program automatically continues to the finish screen.

### Finish screen

The finish screen provides a useful image of the plant with each of the analyzed leaves labeled. These labels correspond to the graph, which opens in a second window. The graph shows the leaf angle as a function of the frame count. Because the frequency at which images are captured in a time‐lapse sequence may be different for dissimilar analyses, the *x*‐value is set as frame counts. The user is given the option to export the collected data to a .csv file. The column number in the output data corresponds to the leaf number as shown in the final image screen.
