# Supplementary material for: Leaf Angle eXtractor: A high‐throughput image processing framework for leaf angle measurements in maize and sorghum
Source: Appl Plant Sci. 2020 Sep 10;8(8):e11385. doi: 10.1002/aps3.11385 (PMC7507698; doi:10.1002/aps3.11385)

Kenchanmane Raju et al.—Applications in Plant Sciences 2020 8(8)—Data Supplement S1. Page 1 of 1.
DOI 10.1002/aps3.11385

APPENDIX S1. Camera setup to acquire time-course images from maize and sorghum plants under water deprivation. (A) Image showing the actual 6-MP camera setup at the Beadle Greenhouses, University of Nebraska–Lincoln. (B) Illustration showing the distance between the camera and the pots, and the perpendicular angle made by the axis of the leaf phyllotaxy with the face of the camera.

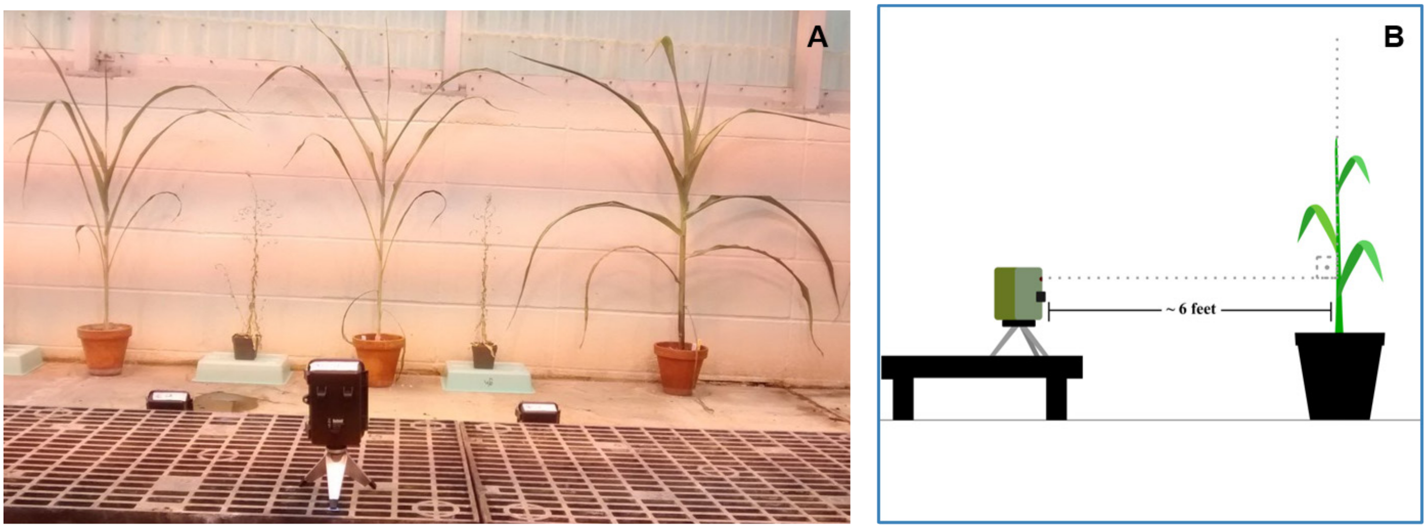

Supplement: Supplementary file 1 — APPENDIX S1. Camera setup to acquire time‐course images from maize and sorghum plants under water deprivation. (A) Image showing the actual 6‐MP camera setup at the Beadle Greenhouses, University of Nebraska–Lincoln. (B) Illustration showing the distance between the camera and the pots, and the perpendicular angle made by the axis of the leaf phyllotaxy with the face of the camera. [file APS3-8-e11385-s001.pdf]
