# Supplementary material for: Leaf Angle eXtractor: A high‐throughput image processing framework for leaf angle measurements in maize and sorghum
Source: Appl Plant Sci. 2020 Sep 10;8(8):e11385. doi: 10.1002/aps3.11385 (PMC7507698; doi:10.1002/aps3.11385)

Kenchanmane Raju et al.—Applications in Plant Sciences 2020 8(8)—Data Supplement S2. Page 1 of 1.
DOI 10.1002/aps3.11385

APPENDIX S2. Stepwise progression of image processing to obtain leaf angle measurements from plant images. (A) Individual plant selected for leaf angle analysis. (B) Image converted to grayscale (in this particular case, blue channel). (C) Enhancing gradient. (D) Inverting color of the picture. (E) Image converted to binary. (F) Image thickened and stalk of the plant emphasized. (G) Unconnected components are bridged, small discontinuities corrected, and image blobs removed. (H) Skeletonized image. (I) Branches are pruned. (J) Determination of leaf angle from the processed image. (Example images shown here are from maize plants grown in the Donald Danforth Plant Science Center, St. Louis, during 2013.)

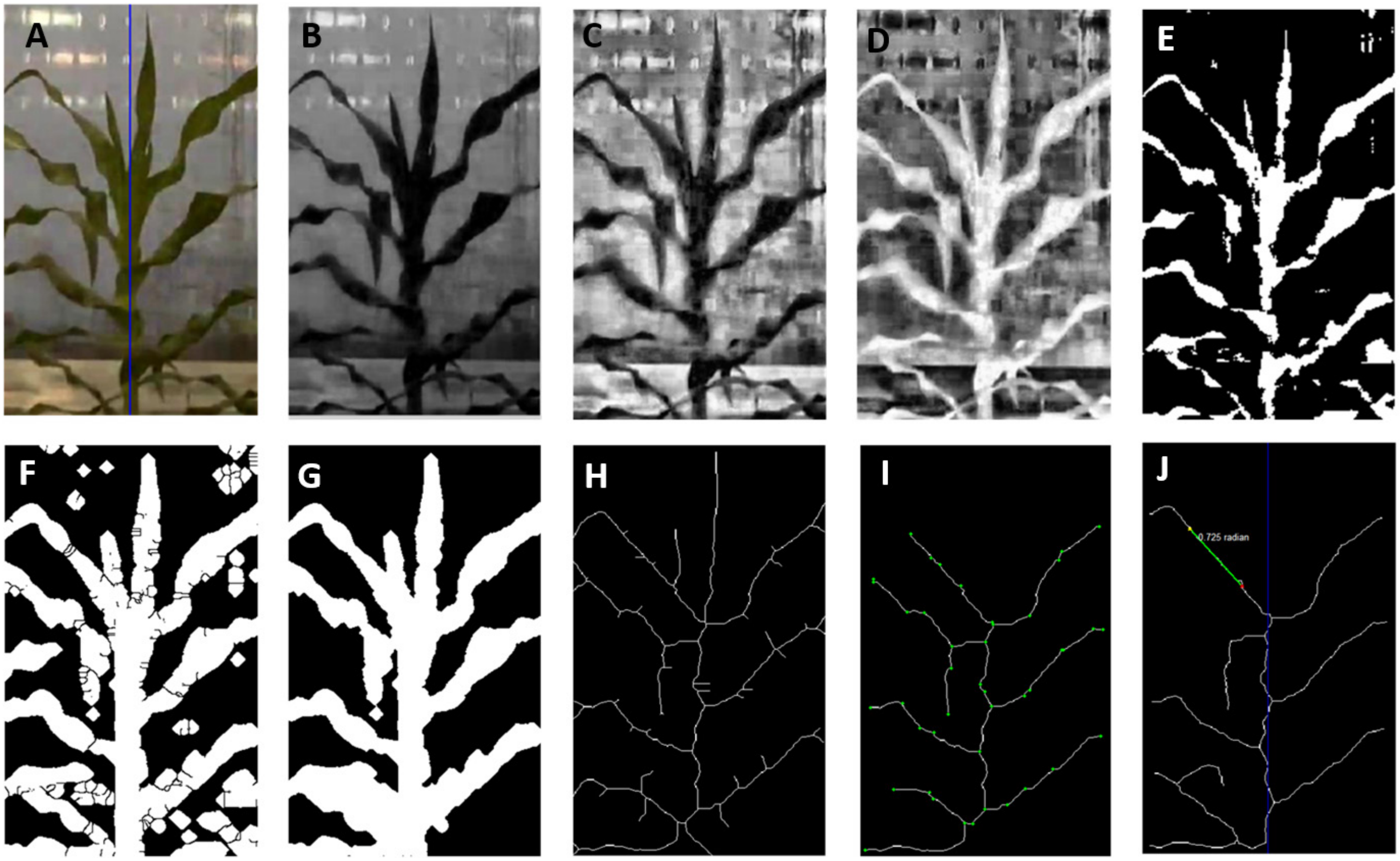

Supplement: Supplementary file 2 — APPENDIX S2. Stepwise progression of image processing to obtain leaf angle measurements from plant images. (A) Individual plant selected for leaf angle analysis. (B) Image converted to grayscale (in this particular case, blue channel). (C) Enhancing gradient. (D) Inverting color of the picture. (E) Image converted to binary. (F) Image thickened and stalk of the plant emphasized. (G) Unconnected components are bridged, small discontinuities corrected, and image blobs removed. (H) Skeletonized image. (I) Branches are pruned. (J) Determination of leaf angle from the processed image. (Example images shown here are from maize plants grown in the Donald Danforth Plant Science Center, St. Louis, during 2013.) [file APS3-8-e11385-s002.pdf]
