# Supplementary material for: Leaf Angle eXtractor: A high‐throughput image processing framework for leaf angle measurements in maize and sorghum
Source: Appl Plant Sci. 2020 Sep 10;8(8):e11385. doi: 10.1002/aps3.11385 (PMC7507698; doi:10.1002/aps3.11385)
Supplement: Supplementary file 3 — APPENDIX S3. Average difference between leaf angle measured using LAX and ground truth measurements. [file APS3-8-e11385-s003.docx]

**APPENDIX S3.** Average difference between leaf angle measured using LAX and ground truth measurements.

| **Species** | **Leaf no.** | **Mean absolute error** |
| --- | --- | --- |
| Maize_1 | Leaf_1 | 3.84 |
|  | Leaf_2 | 1.87 |
|  | Leaf_3 | 2.53 |
| Maize_2 | Leaf_1 | 3.46 |
|  | Leaf_2 | 2.76 |
|  | Leaf_3 | 5.06 |
|  | Leaf_4 | 3.38 |
|  | Leaf_5 | 11.18 |
| Maize_3 | Leaf_1 | 6.94 |
|  | Leaf_2 | 2.1 |
|  | Leaf_3 | 5.56 |
|  | Leaf_4 | 1.49 |
| Sorghum_1 | Leaf_1 | 1.28 |
|  | Leaf_2 | 4.39 |
|  | Leaf_3 | 1.97 |
|  | Leaf_4 | 3.12 |
| Sorghum_2 | Leaf_1 | 1.12 |
|  | Leaf_2 | 4.05 |
|  | Leaf_3 | 4.03 |
|  | Leaf_4 | 2.14 |
| Sorghum_3 | Leaf_1 | 1.37 |
|  | Leaf_2 | 3.44 |
|  | Leaf_3 | 2.08 |
|  | Leaf_4 | 5.95 |
