# Supplementary material for: Leaf Angle eXtractor: A high‐throughput image processing framework for leaf angle measurements in maize and sorghum
Source: Appl Plant Sci. 2020 Sep 10;8(8):e11385. doi: 10.1002/aps3.11385 (PMC7507698; doi:10.1002/aps3.11385)

DOI 10.1002/aps3.11385

APPENDIX S4. Differential phenotypic response to drought stress in sorghum (A–C) and maize (D–F). (A) Six-week-old sorghum plant (Btx623). (B) Sorghum plant nine days after water deprivation. (C) Sorghum plant showing leaf rolling symptoms 11 days after water deprivation. (D) Six-week-old maize plant (B73). (E) Maize plant nine days after water deprivation showing signs of leaf rolling. (F) Maize plant showing leaf wilting after 11 days of water deprivation.

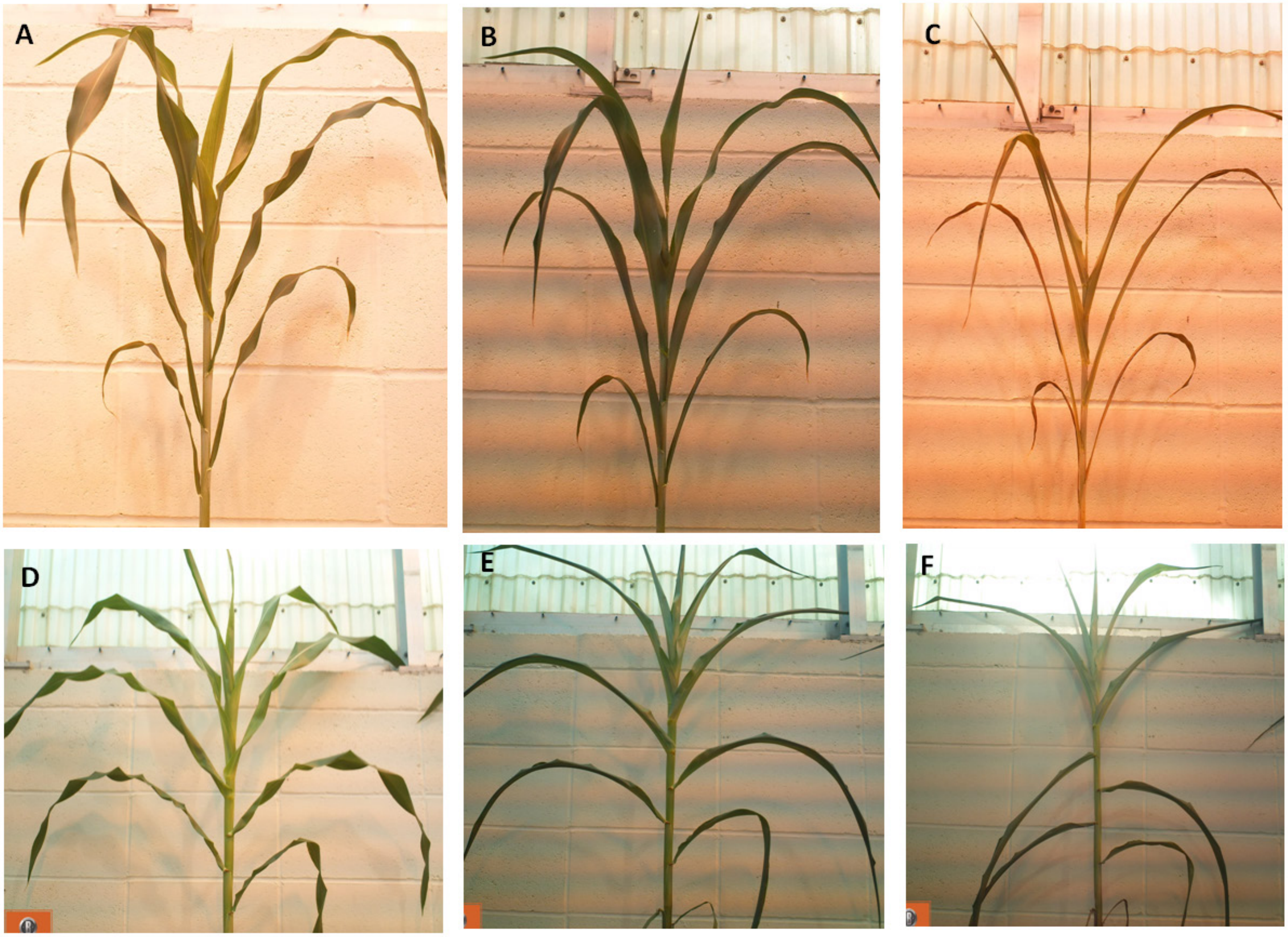

Supplement: Supplementary file 4 — APPENDIX S4. Differential phenotypic response to drought stress in sorghum (A–C) and maize (D–F). (A) Six‐week‐old sorghum plant (Btx623). (B) Sorghum plant nine days after water deprivation. (C) Sorghum plant showing leaf rolling symptoms 11 days after water deprivation. (D) Six‐week‐old maize plant (B73). (E) Maize plant nine days after water deprivation showing signs of leaf rolling. (F) Maize plant showing leaf wilting after 11 days of water deprivation. [file APS3-8-e11385-s004.pdf]
