# Supplementary material for: Leaf Angle eXtractor: A high‐throughput image processing framework for leaf angle measurements in maize and sorghum
Source: Appl Plant Sci. 2020 Sep 10;8(8):e11385. doi: 10.1002/aps3.11385 (PMC7507698; doi:10.1002/aps3.11385)

DOI 10.1002/aps3.11385

APPENDIX S5. Consistent wilting of the upper leaf with clearly visible collar across replicates. (A–C) Maize plant 9-M2, showing wilting of leaf after day 11 of water deprivation. (D–F) Maize plant 10-M3, showing wilting of the same leaf after day 11 of water deprivation. The blue arrow indicates the leaf that showed wilting in both maize replicate through the time-course images.

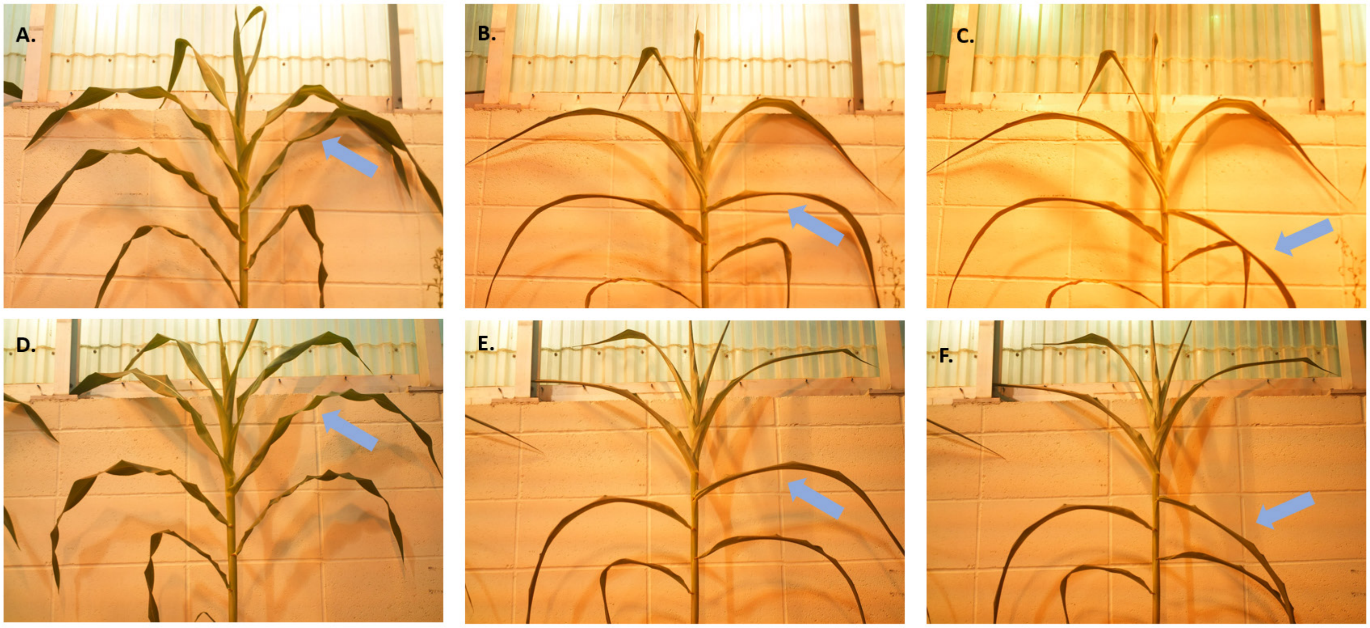

Supplement: Supplementary file 5 — APPENDIX S5. Consistent wilting of the upper leaf with clearly visible collar across replicates. (A–C) Maize plant 9‐M2, showing wilting of leaf after day 11 of water deprivation. (D–F) Maize plant 10‐M3, showing wilting of the same leaf after day 11 of water deprivation. The blue arrow indicates the leaf that showed wilting in both maize replicate through the time‐course images. [file APS3-8-e11385-s005.pdf]
