# Supplementary material for: Leaf Angle eXtractor: A high‐throughput image processing framework for leaf angle measurements in maize and sorghum
Source: Appl Plant Sci. 2020 Sep 10;8(8):e11385. doi: 10.1002/aps3.11385 (PMC7507698; doi:10.1002/aps3.11385)

DOI 10.1002/aps3.11385

APPENDIX S6. Sample screens showing LAX framework usage. (A) Graphical user interface (GUI) welcome screen for the LAX framework obtained after running new_wilt_gui.m function in MATLAB. (B) Selection of the first image in the series. (All images belonging to a series stored in a folder can be loaded at once.) (C) Selecting the stalk of the plant by clicking the cursor at the center of the plant stalk. (D) Adjusting the width and the height of the plant image suitable for leaf angle measurements. (E) Selection of leaves for leaf angle measurement. Rectangles must be drawn close to the leaf–stalk junction without touching the stalk. Care must be taken while drawing the rectangle so that the entire change in the leaf angle can be captured in the drawn rectangle (as shown in H). (F) Leaf angle measurements will be recorded for as many leaves as selected by the user. (G) Thresholding can be adjusted by moving the slider or inputting threshold values. (H) When thresholding is completed for the last image in the series, the screen shows both the first and the last image and the rectangles drawn to select the leaves. (I) Clicking 'Start analysis' begins the analysis of each leaf for angle measurements. (J) The final screen shows the plant image with rectangle boxes and leaf number. Clicking the 'Export Data' icon at the bottom outputs leaf angle measurements for the selected leaves as a .csv file.

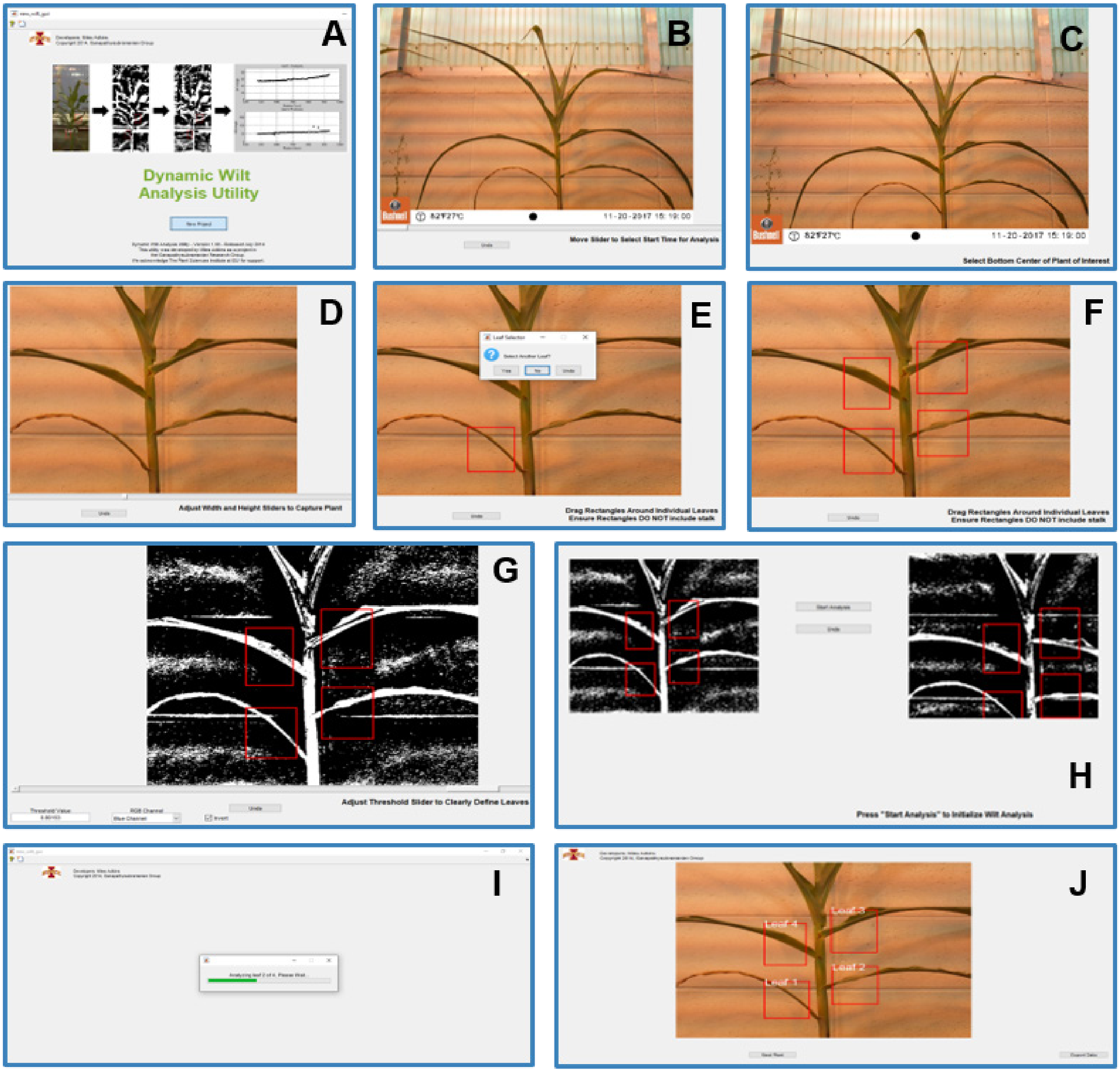

Supplement: Supplementary file 6 — APPENDIX S6. Sample screens showing LAX framework usage. (A) Graphical user interface (GUI) welcome screen for the LAX framework obtained after running new_wilt_gui.m function in MATLAB. (B) Selection of the first image in the series. (All images belonging to a series stored in a folder can be loaded at once.) (C) Selecting the stalk of the plant by clicking the cursor at the center of the plant stalk. (D) Adjusting the width and the height of the plant image suitable for leaf angle measurements. (E) Selection of leaves for leaf angle measurement. Rectangles must be drawn close to the leaf–stalk junction without touching the stalk. Care must be taken while drawing the rectangle so that the entire change in the leaf angle can be captured in the drawn rectangle (as shown in H). (F) Leaf angle measurements will be recorded for as many leaves as selected by the user. (G) Thresholding can be adjusted by moving the slider or inputting threshold values. (H) When thresholding is completed for the last image in the series, the screen shows both the first and the last image and the rectangles drawn to select the leaves. (I) Clicking ‘Start analysis’ begins the analysis of each leaf for angle measurements. (J) The final screen shows the plant image with rectangle boxes and leaf number. Clicking the ‘Export Data’ icon at the bottom outputs leaf angle measurements for the selected leaves as a .csv file. [file APS3-8-e11385-s006.pdf]
